# Hippuristanol Reduces the Viability of Primary Effusion Lymphoma Cells both *in Vitro* and *in Vivo*

**DOI:** 10.3390/md11093410

**Published:** 2013-09-06

**Authors:** Chie Ishikawa, Junichi Tanaka, Harutaka Katano, Masachika Senba, Naoki Mori

**Affiliations:** 1 Department of Microbiology and Oncology, Graduate School of Medicine, University of the Ryukyus, 207 Uehara, Nishihara, Okinawa 903-0215, Japan; E-Mail: chiezo@lab.u-ryukyu.ac.jp; 2 Transdisciplinary Research Organization for Subtropics and Island Studies, University of the Ryukyus, 1 Senbaru, Nishihara, Okinawa 903-0213, Japan; 3 Department of Chemistry, Biology and Marine Science, University of the Ryukyus, 1 Senbaru, Nishihara, Okinawa 903-0213, Japan; E-Mail: jtanaka@sci.u-ryukyu.ac.jp; 4 Department of Pathology, National Institute of Infectious Diseases, 1-23-1 Toyama, Shinjuku, Tokyo 162-8640, Japan; E-Mail: katano@nih.go.jp; 5 Department of Pathology, Institute of Tropical Medicine, Nagasaki University, 1-12-4 Sakamoto, Nagasaki 852-8523, Japan; E-Mail: mikiyo@nagasaki-u.ac.jp

**Keywords:** hippuristanol, PEL, AP-1, STAT3, Akt

## Abstract

Primary effusion lymphoma (PEL) caused by Kaposi’s sarcoma-associated herpesvirus (also known as human herpesvirus-8) shows serious lymphomatous effusion in body cavities. PEL is difficult to treat and there is no standard treatment strategy. Hippuristanol is extracted from Okinawan coral *Isis hippuris*, and inhibits translational initiation by blocking eukaryotic initiation factor 4A, an ATP-dependent RNA helicase, binding to mRNA. Recently, there has been much interest in targeting translation initiation as an anticancer therapy. Here, we show that treatment of PEL cell lines with hippuristanol resulted in cell cycle arrest at G_1_ phase, and induced caspases activation and apoptosis. Hippuristanol also reduced the expression of cyclin D2, CDK2, CDK4, CDK6 and prosurvival XIAP and Mcl-1 proteins. Activation of activator protein-1, signal transducers and activators of transcription protein 3 and Akt pathways plays a critical role in the survival and growth of PEL cells. Hippuristanol suppressed the activities of these three pathways by inhibiting the expression of JunB, JunD, c-Fos, signal transducers and activators of transcription protein 3 and Akt proteins. In a xenograft mouse model that showed ascites and diffused organ invasion of PEL cells, treatment with hippuristanol significantly inhibited the growth and invasion of PEL cells compared with untreated mice. The results of the *in vitro* and *in vivo* experiments underline the potential usefulness of hippuristanol in the treatment of PEL.

## 1. Introduction

The lymphoproliferative disorder, primary effusion lymphoma (PEL), afflicts mainly patients infected with human immunodeficiency virus, and exclusively involves body cavities such as the peritoneal, pleural and pericardial cavities [1,2]. PEL is caused by clonal expansion of Kaposi’s sarcoma-associated herpesvirus (KSHV; also known as human herpesvirus-8) infected B cells [1,2]. Patients with PEL present with lymphomatous effusions within body cavities [1,2]. This type of lymphoma is generally aggressive, rapidly progressing and resistant to conventional chemotherapy, with a median survival time of six months and a one-year overall survival rate of 40% [3], emphasizing the need for new therapies. A number of constitutively activated signaling pathways play critical roles in the survival and growth of PEL cells. These include nuclear factor kappa B (NF-κB), activator protein-1 (AP-1), signal transducers and activators of transcription protein (STAT) and phosphatidylinositol-3-kinase (PI3K)/Akt pathways [4,5,6,7]. KSHV is a master in altering these pathways in favor of its survival [8]. 

Hippuristanol, a steroid isolated from coral *Isis hippuris*, has been identified as a selective and potent inhibitor of RNA helicase, eukaryotic initiation factor 4A, and RNA-binding activity [9]. The eukaryotic initiation factor 4A plays a key role in the recruitment of ribosomes to mRNA templates during the initiation of eukaryotic translation. Recently, there has been much interest in targeting translation initiation as an anticancer therapy, because deregulated translation initiation is implicated extensively in cancer initiation and progression [10]. 

In the present study, we investigated the effects of hippuristanol on the proliferation and apoptosis of cultured human PEL cells and analyzed the mechanisms of such effects. We also assessed the *in vivo* effects of hippuristanol in immunodeficient SCID mice inoculated with PEL cells. The results provide insights into the molecular target of hippuristanol in PEL cells and the anticancer mechanism of hippuristanol against PEL cells.

## 2. Results

### 2.1. Hippuristanol Dose-Dependently Inhibits PEL Cell Viability

**Figure 1:**
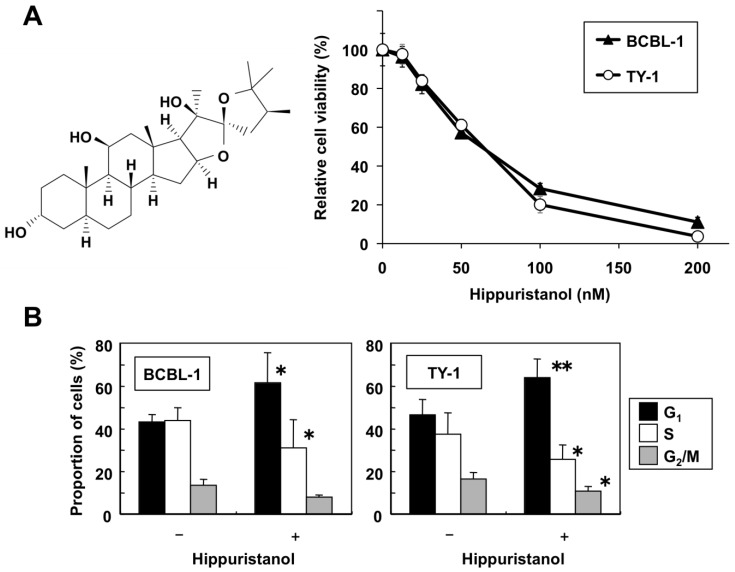
Hippuristanol reduces viability and induces cell cycle arrest of primary effusion lymphoma (PEL) cells. (**A**) *Left:* Structure of hippuristanol. *Right:* Hippuristanol reduced PEL cell viability. PEL cell lines were treated with the indicated concentrations of hippuristanol for 24 h. The cell viability was determined by WST-8. Data are mean ± SD percentage values of triplicate experiments compared with untreated cells; (**B**) Hippuristanol causes PEL cell cycle arrest. PEL cells were treated with hippuristanol (200 nM) for 24 h and analyzed by flow cytometry. The percentages of cells in the G_1_, S and G_2_/M phases were calculated using MultiCycle software. Data are mean ± SD percentage values of cells at various phases of the cell cycle (*n* = 3). * *P* < 0.05, ** *P* < 0.01, compared with control cells.

First, we examined the effects of hippuristanol on PEL cell viability. Two PEL cell lines (BCBL-1 and TY-1), two KSHV-uninfected lymphoma B cell lines (BJAB and Ramos), and peripheral blood mononuclear cells (PBMCs) from three healthy volunteers were cultured for 24 h in the presence of various concentrations of hippuristanol, and their viability was analyzed by water-soluble tetrazolium (WST)-8 assays. Figure 1A shows that increasing the concentration of hippuristanol from 12.5 to 200 nM resulted in further suppression of cell viability and that this effect was dose-dependent in the two PEL cell lines. The estimated 50% inhibitory concentration (IC_50_) values for BCBL-1 and TY-1 were 62 and 55 nM, respectively. In contrast, the IC_50_ values for BJAB and Ramos were 175 and 104 nM, respectively. We reported previously that the IC_50_ values for five human T cell leukemia virus type 1-infected T cell lines ranged from 189 to 329 nM [11]. Thus, PEL cell lines were considered more sensitive than T cell lines and KSHV-uninfected lymphoma B cell lines to hippuristanol. On the other hand, PBMCs from healthy volunteers were resistant to hippuristanol with IC_50_ of >1021 nM [11]. These results suggest that hippuristanol is less cytotoxic to normal cells than PEL cells, and most effectively inhibited cell survival of PEL cells at low nanomolar concentrations.

### 2.2. Effects of Hippuristanol on PEL Cell Cycle and Apoptosis

In following experiments, we determined the mechanism of the suppressive effects of hippuristanol on PEL cell viability. The effect of hippuristanol on cell cycle progression was investigated by flow cytometry analysis after propidium iodide staining. Hippuristanol accumulated cells in sub-G_1_ phase (from 3.9% and 2.9% of control BCBL-1 and TY-1 cells to 17.4% and 13.9% of treated BCBL-1 and TY-1 cells, respectively). A cell cycle profile was then created by using selective gating excluding sub-G_1_ population. As shown in Figure 1B, hippuristanol increased the G_1_ population of PEL cells, compared with the control. This increase was accompanied by a concomitant decrease in the S phase and G_2_/M phase cell populations. These results indicate that the inhibitory effects of hippuristanol on PEL cell viability were due to cell cycle arrest at G_1_ phase. 

**Figure 2:**
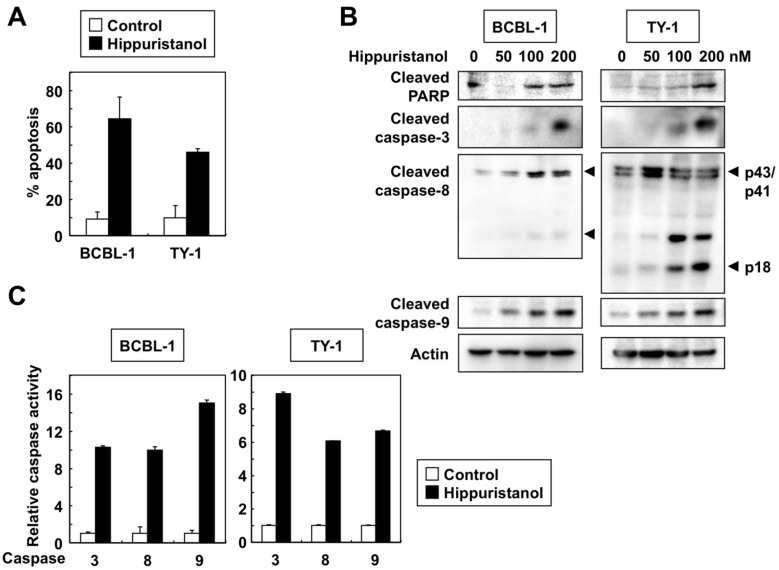
Hippuristanol induces apoptosis of PEL cells. (**A**) PEL cells were cultured in the presence or absence of hippuristanol (200 nM) for 24 h and apoptosis was determined by Apo2.7 staining. Data are mean ± SD; (**B**) Immunoblot analysis of caspases and PARP cleavage. PEL cells were treated with the indicated concentrations of hippuristanol for 24 h, and then subjected to Western blotting. Arrowheads indicate the cleaved form of caspase-8; (**C**) Changes in caspase-3, -8 and -9 activities in PEL cells. Cells were cultured in the presence or absence of hippuristanol (200 nM) for 24 h before harvesting. The activity of the indicated caspases was measured using the colorimetric caspase assay and labeled caspase substrates. Caspase activity in untreated cells was defined as 1. Data are mean ± SD of three experiments.

Since cells with sub-G_1_ DNA content were considered apoptotic, we determined the extent of apoptosis in hippuristanol-treated PEL cells by using Apo2.7 staining. Apo2.7 specifically detects the 38-kDa mitochondrial membrane antigen 7A6 expressed on the mitochondrial outer membrane during apoptosis [12]. As shown in Figure 2A, the addition of 200 nM hippuristanol to cultures of PEL cells for 24 h resulted in apoptosis of these cells. Next, we studied the role of caspases in this process by determining cleavage of endogenous caspases. Western blot analyses carried out after treatment of PEL cells with hippuristanol showed increased levels of activated cleaved forms of caspase-3, -8 and -9, and that such increases were hippuristanol dose-dependent (Figure 2B). Caspase-3 has several substrate proteins, and the DNA damage repair enzyme polyadenosin-5′-diphosphate-ribose polymerase (PARP) is a major substrate [13]. The cleaved PARP was present as an active form and its production level was hippuristanol dose-dependent. Control experiments showed no change in the expression of the structural protein actin after the addition of hippuristanol up to 200 nM.

Immunoblotting allowed us to examine the processing of caspases, but did not indicate whether the cleavage products were enzymatically active. Therefore, we used colorimetric assays to determine caspase-3, -8 and -9 activities based on cleavage of caspase-specific-labeled substrates. Hippuristanol activated caspase-3, -8 and -9 in PEL cells (Figure 2C). The results of the above experiments confirmed that caspase activation mediates hippuristanol-induced apoptosis of PEL cells.

### 2.3. Effects of Hippuristanol on Expression of Cell Cycle and Apoptosis Regulatory Proteins in PEL Cells

**Figure 3:**
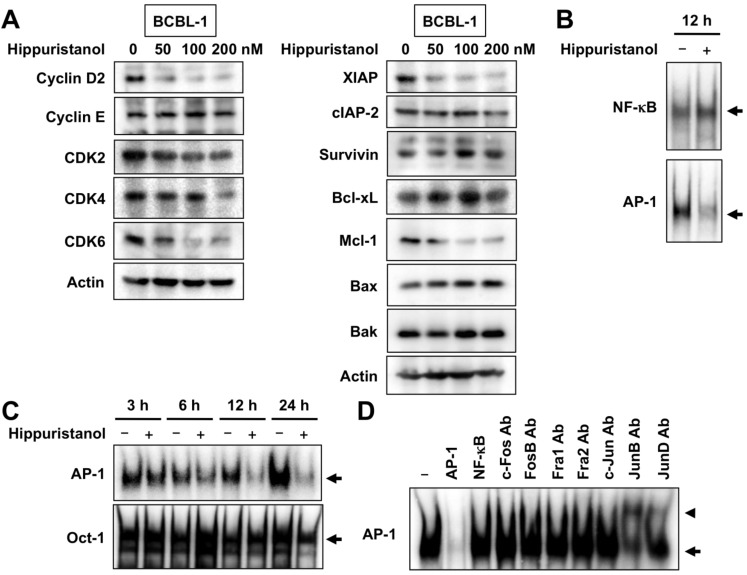
Hippuristanol induces downregulation of cell cycle and apoptosis regulatory proteins and suppresses AP-1 signaling in PEL cells. (**A**) Effects of hippuristanol on the expression of cell cycle (left panels) and apoptosis regulatory proteins (right panels). BCBL-1 cells were treated with the indicated concentrations of hippuristanol for 24 h and then harvested. Total cell lysates were prepared and equal amounts of protein were subjected to Western blot analysis; (**B** and **C**) Hippuristanol inhibits AP-1 activation. BCBL-1 cells were treated with hippuristanol (200 nM) for the indicated time periods and assessed for AP-1, NF-κB and Oct-1 DNA-binding activity by EMSA using each specific oligonucleotide probe; (**D**) Competition and supershift assays were performed by adding the indicated competitor oligonucleotides or antibodies (Ab), to nuclear extracts from BCBL-1 cells.

The cell cycle process in eukaryotes is orchestrated by the function of a family of protein kinase complexes. Each complex is composed minimally of cyclins that bind to CDK to form active cyclin-CDK complexes. We next examined the effects of hippuristanol on cell cycle regulatory molecules operative in the G_1_ phase of the cell cycle in PEL cells. Western blot analysis showed that treatment of BCBL-1 cells with hippuristanol resulted in a significant and dose-dependent decrease in the protein expression of cyclin D2, CDK2, CDK4 and CDK6, but not cyclin E (Figure 3A).

We also examined whether the effects of hippuristanol on apoptosis is mediated through the modulation of expression of inhibitors of apoptosis. Hippuristanol caused a dose-dependent downregulation of XIAP and Mcl-1, but had no effect on the expression of cIAP-2, survivin and Bcl-xL (Figure 3A). Furthermore, hippuristanol did not alter the expression of pro-apoptotic proteins, Bax and Bak. These results suggest the possible involvement of XIAP and Mcl-1 proteins in hippuristanol-induced apoptosis.

### 2.4. Hippuristanol Efficiently Blocks Constitutive Activation of AP-1 in PEL Cells

**Figure 4:**
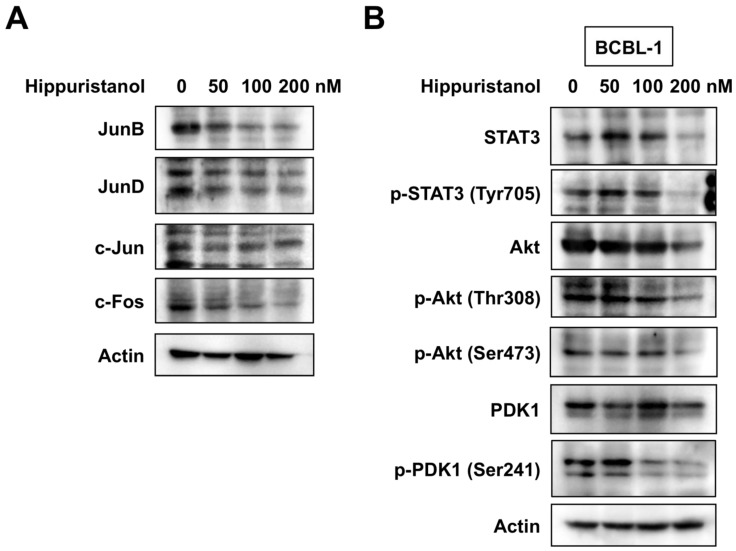
Hippuristanol inhibits the constitutively active AP-1, STAT3 and Akt in PEL cells. (**A**) Hippuristanol reduced AP-1 family proteins, JunB, JunD and c-Fos; (**B**) Hippuristanol inhibited the activation of STAT3 and Akt. BCBL-1 cells were treated with various doses of hippuristanol for 24 h, and cell lysates were prepared. Equal amounts of protein from each sample were separated and immunoblotted with the indicated antibodies.

Several reports have suggested that NF-κB and AP-1 can act as survival factors and are required for the proliferation of PEL cells [4,7]. Because NF-κB and AP-1 are constitutively active in PEL cells [4,7], we examined whether hippuristanol inhibits NF-κB and AP-1 activation using electrophoretic mobility shift assay (EMSA). Treatment with 200 nM hippuristanol suppressed the DNA-binding activities of AP-1 but not NF-κB in BCBL-1 cells (Figure 3B). The suppression was specific to AP-1 and was not due to cell death because no significant change in binding activity of Oct-1 was observed after treatment of cells with hippuristanol (Figure 3C). The specificity of the gel retardation was demonstrated in cold competition experiments. Excess of the cold AP-1 probe abrogated the band, whereas excess of the cold NF-κB probe had no effect (Figure 3D). We next analyzed AP-1 subcomponents by supershift assays using BCBL-1 cells. Supershift analyses showed that AP-1 band was composed of JunB and JunD (Figure 3D). We then examined the effect of hippuristanol on the expression of JunB and JunD proteins in BCBL-1 cells. When incubated with hippuristanol for 24 h, BCBL-1 cells displayed decreased expression of JunB and JunD, and the decrease was hippuristanol dose-dependent (Figure 4A). c-Jun, the most potent transcription factor in AP-1 family, forms heterodimer with c-Fos. The KSHV-encoded latency-associated nuclear antigen induces binding of a c-Jun-Fos heterodimer to the AP-1 response element [14]. We also examined the effect of hippuristanol on the expression of c-Jun and c-Fos proteins. As shown in Figure 4A, hippuristanol reduced the expression of c-Fos, but not c-Jun. These results suggest that hippuristanol inhibits AP-1 activation through depletion of JunB, JunD and c-Fos.

### 2.5. Hippuristanol Inhibits Activation of STAT3 and Akt in PEL Cells

Aberrant STAT3 and Akt activation is also found in PEL cells [5,6]. The next series of experiments examined the inhibitory effect of hippuristanol on STAT3 and Akt activation in BCBL-1 cells using Western blot analysis. Phosphorylation of STAT3 and Akt was detected in BCBL-1 cells, and hippuristanol suppressed the levels of total and phosphorylated STAT3 and Akt proteins (Figure 4B). Phosphatidylinositol 3-kinase (PI3K) catalyzes the phosphorylation of the 3-hydroxyl position of phosphatidylinositol 4,5-diphosphate (PIP2) to phosphatidylinositol 3,4,5-triphosphate (PIP3). PIP3 facilitates the phosphorylation of Akt at Thr308 by 3-phosphoinositide-dependent protein kinase 1 (PDK1) [15]. Phosphorylation on the activation loop Ser241 by autophosphorylation is necessary for PDK1 activity [16]. The phosphorylation level of PDK1 was also examined at this time point. Hippuristanol inhibited the phosphorylation of PDK1 in a dose-dependent manner (Figure 4B), but had no effect on PDK1 total protein level. These results suggest that hippuristanol inhibits constitutive activation of STAT3 and Akt in PEL cells, and that Akt inhibition is due at least in part to PDK1 dephosphorylation. 

### 2.6. *In Vivo* Effects of Hippuristanol in Immunodeficient Mice Inoculated with PEL Cells

**Figure 5:**
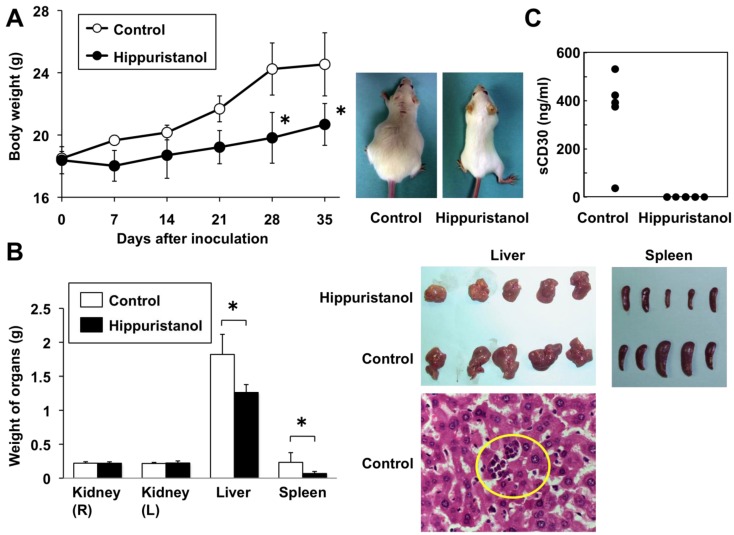
Treatment of SCID mice with hippuristanol suppresses the development of PEL *in vivo*. (**A**) *Left:* Body weight of mice inoculated with BCBL-1 cells and treated or untreated with hippuristanol. Data are mean ± SD of five mice. *Right:* Photographs of hippuristanol-treated and -untreated ascites-bearing mice five weeks after intraperitoneal inoculation of BCBL-1; (**B**) Metastasis of PEL cells into the liver and spleen in BCBL-1 inoculated mice. *Left:* Weight of the indicated organs in mice inoculated with BCBL-1 cells and treated or untreated with hippuristanol. Data are mean ± SD of five mice. * *P* < 0.05, compared with the vehicle-treated group. *Top* *Right:* Photographs of the livers and spleens in hippuristanol- and vehicle-treated mice. *Bottom Right*: H&E staining was performed to detect BCBL-1 cells in the liver in mice untreated with hippuristanol. Original magnification, ×400; (**C**) Serum levels of sCD30 in hippuristanol-treated and -untreated mice. The sCD30 concentrations were measured by ELISA.

The above *in vitro* experiments demonstrated the efficacy of hippuristanol against PEL cells. In the next series of experiments, we tested the effects of hippuristanol in immunodeficient mice. BCBL-1 cells were injected intraperitoneally into the SCID mice and hippuristanol or vehicle was administered intraperitoneally every day. BCBL-1 produced massive ascites within 35 days of inoculation. Vehicle-treated mice developed massive abdominal distention, whereas hippuristanol-treated mice appeared normal and healthy. The body weight of hippuristanol-treated mice was significantly less than that of vehicle-treated mice at 28 and 35 days (Figure 5A). Evaluation of PEL cell infiltration in body organs on day 35 by hematoxylin and eosin (H&E) staining showed infiltration of BCBL-1 cells in the liver and spleen of vehicle-treated mice (Figure 5B). In contrast, the infiltration of BCBL-1 cells into the liver and spleen in hippuristanol-treated mice was not observed. The weight of the livers and spleens was significantly lower in hippuristanol-treated mice than in vehicle-treated mice (Figure 5B). No apparent adverse effects were observed in mice treated with hippuristanol.

PEL cells are known to express CD30, a 120-kDa type I surface glycoprotein [1] and soluble CD30 (sCD30) is produced by PEL cells [17]. The serum level of sCD30 appears to be a useful biological tumor marker for the diagnosis and management of PEL [17]. Treatment of mice with hippuristanol significantly reduced serum sCD30 to undetectable levels, whereas that of vehicle-treated mice was 352 ± 186 ng/mL (*n* = 5) (Figure 5C). These data indicate that hippuristanol significantly inhibits the growth and infiltration of PEL cells *in vivo*. Collectively, these results demonstrated that hippuristanol is effective against PEL cells. 

## 3. Discussion

In the present study, we investigated the effects of a naturally occurring eukaryotic translation initiation inhibitor, hippuristanol, on PEL cells, both *in vitro* and *in vivo*, and showed that hippuristanol is cytotoxic to these cells and this action is mediated through inhibition of the AP-1, STAT3 and Akt pathways and depletion of JunB, JunD, c-Fos, STAT3 and Akt. Since hippuristanol did not significantly alter the ratios of phosphorylated STAT3 *versus* total STAT3 and phosphorylated Akt *versus* total Akt, the decrease in phosphorylated STAT3 and Akt is possibly due to the downregulation of total STAT3 and Akt, rather than their dephosphorylation. In this study, we found that hippuristanol disrupted the phosphorylation of PDK1 in PEL cells. We also showed that hippuristanol reduced the expression of cyclin D2, CDK2, CDK4, CDK6, XIAP and Mcl-1 proteins. Cyclin D2 promoter contains AP-1 site [18]. Constitutively activated STAT3 is known to participate in oncogenesis through upregulation of STAT3 target genes encoding cyclin D2 [19] and apoptosis inhibitors such as XIAP, which is known to suppress cell death by inhibiting caspase-3 and caspase-9 [20]. Furthermore, Akt interacts with and phosphorylates XIAP, leading to inhibition of ubiquitination of XIAP [21]. A link between STAT3 activation and regulation of Bcl-2 family protein expression has been demonstrated [22]. Inhibition of STAT3 signaling resulted in apoptosis and decreased Mcl-1 expression [22]. Mcl-1 is also a PI3K-regulated protein [23]. In addition, STAT3 can regulate the AP-1 family protein, JunB [24]. Thus, AP-1, STAT3 and Akt can collaborate in PEL, depending on various of survival factors. Although hippuristanol-induced downregulation of cell cycle and apoptosis regulatory proteins depends on direct inhibition of translation, we speculate that this agent blocks the AP-1, STAT3 and Akt signaling pathways, resulting in reduced expression of their downstream effectors. 

It is known that hippuristanol delays poliovirus replication in infected cells [9]. Therefore, we investigated whether hippuristanol affects KSHV lytic replication in PEL cells. The expression of two viral genes known as lytic genes (ORF50 and ORFK9) and three viral genes known as latent genes (ORF72, ORF73 and ORFK13) was examined in TY-1 cells treated with hippuristanol by reverse transcription-polymerase chain reaction. However, there was no significant change in the expression levels of lytic and latent genes after treatment (data not shown), suggesting that hippuristanol does not induce viral reactivation.

*In vivo* experiments showed that untreated PEL-xenografted mice tended to be heavier, both in appearance and weight. The mean body weight and weight of livers and spleens were significantly higher in the control group, which was likely due to effusion in body cavities and organ invasion. Treatment with hippuristanol reduced serum levels of the surrogate marker sCD30 to undetectable levels, compared with relatively high levels in untreated mice. Although there is still the possibility that hippuristanol directly inhibits CD30 mRNA translation, treatment with hippuristanol could induce cell death of PEL cells, a major source for sCD30, resulting in a decrease in serum sCD30. The significant reduction of ascites and organ invasion with no apparent adverse effects in mice suggests that hippuristanol could add a new direction in the treatment of refractory PEL. 

## 4. Experimental Section

### 4.1. Cell Lines and Reagents

Human KSHV-infected PEL cell lines, BCBL-1 [25] and TY-1 [26], and KSHV-uninfected lymphoma B cell lines, BJAB and Ramos, were maintained in Roswell Park Memorial Institute (RPMI) 1640 medium supplemented with 10% heat inactivated fetal bovine serum, penicillin (50 U/mL) and streptomycin (50 µg/mL) in a humidified incubator. Hippuristanol was extracted from the gorgonian *I. hippuris* as described previously [9]. Final purification of hippuristanol was done by HPLC up to 97%. Its purity was confirmed by 1H NMR spectrum. Antibodies to cleaved PARP, cleaved caspase-3, cleaved caspase-8, cleaved caspase-9, survivin, Bcl-xL, Bax, Bak, c-Jun, Akt, phospho-Akt (Thr308), phospho-Akt (Ser473), PDK1, phospho-PDK1 (Ser241), STAT3 and phospho-STAT3 (Tyr705) were purchased from Cell Signaling Technology (Beverly, MA, USA). Antibodies to cyclin E, CDK2, CDK4, CDK6 and actin were purchased from NeoMarkers (Fremont, CA, USA). The antibody to XIAP was obtained from Medical & Biological Laboratories (Nagoya, Japan). Antibodies to cyclin D2, cIAP-2, Mcl-1, JunB, JunD and c-Fos were purchased from Santa Cruz Biotechnology (Santa Cruz, CA, USA). 

### 4.2. Assesement of Cell Viability and Apoptosis

Cell viability was determined by color reaction with WST-8 (Wako Pure Chemical Industries, Osaka, Japan). Mitochondrial dehydrogenase cleavage of WST-8 to formazan dye provided a measure of cell viability. Briefly, 1 × 10^5^ cells/mL were incubated in triplicate in a 96-well microculture plate in the presence of different concentrations of hippuristanol for 24 h. Subsequently, WST-8 was added to each well. After 4 h of additional incubation, absorption values at 450 nm were determined with an automatic microplate reader. Values were normalized to untreated control samples. The IC_50_ value was calculated by dotting the data points to a logistic curve using the CalcuSym software. Apoptotic events in cells were detected by staining with phycoerythrin-conjugated Apo2.7 antibody (Beckman Coulter, Marseille, France), which specifically detects the 38-kDa mitochondrial membrane antigen 7A6 [12], and analyzed by flow cytometry.

### 4.3. Cell Cycle Analysis

Log phase growing cells were treated with hippuristanol (200 nM) in complete culture medium for 24 h. Cell cycle analysis was performed with the CycleTEST PLUS DNA reagent kit (Becton Dickinson Immunocytometry Systems, San Jose, CA, USA). Cell suspensions were analyzed by flow cytometry. The population of cells in each cell cycle phase was determined with MultiCycle software. 

### 4.4. *In Vitro* Measurement of Caspase Activity

Caspase activity was measured using colorimetric caspase assay kits (Medical & Biological Laboratories). Cell extracts were recovered using the cell lysis buffer supplied with the kit and assessed for caspase-3, -8 and -9 activities using colorimetric probes. The assay kits are based on detection of chromophore ρ-nitroanilide after cleavage from caspase-specific labeled substrates. Colorimetric readings were performed in an automated microplate reader.

### 4.5. Western Blotting

Cells were treated with various concentrations of hippuristanol in complete medium for 24 h. Cells were lysed in a buffer containing 62.5 mM Tris-HCl (pH 6.8), 2% sodium dodecyl sulfate, 10% glycerol, 6% 2-mercaptoethanol and 0.01% bromophenol blue. For Western blotting, 20 µg protein was resolved over polyacrylamide gels and transferred to a polyvinylidene difluoride membrane, and probing with the specific antibodies. Detection was performed using Amersham Biosciences enhanced chemiluminescence kit (Piscataway, NJ, USA). 

### 4.6. Electrophoretic Mobility Shift Assay (EMSA)

Nuclei were extracted from cells using the method described previously [27] with some modifications. EMSA was conducted as described by Mori and Prager [28]. Briefly, nuclear extracts (5 µg) were incubated with ^32^P-labeled probes, followed by separation of the DNA-protein complex from free oligonucleotides on 4% polyacrylamide gel. The sense and antisense synthetic oligonucleotides, containing the AP-1 element from interleukin-8 gene (5′-gatcGTGATGACTCAGGTT-3′) and the NF-κB element from interleukin-2 receptor α chain gene (5′-gatcCGGCAGGGGAATCTCCCTCTC-3′), were annealed and the resultant probes were used. The oligonucleotide 5′- gatcTGTCGAATGCAAATCACTAGAA-3′, containing the consensus sequence of the octamer binding motif, was used to identify specific binding of the transcription factor Oct-1, which regulates the transcription of a number of so-called housekeeping genes. The above underlined sequences represent the AP-1, NF-κB and Oct-1 binding sites, respectively. Cold-competition experiments were performed with excess of cold AP-1 or NF-κB oligonucleotides. For supershift assays, the prepared extracts were preincubated with antibodies against c-Fos, FosB, Fra1, Fra2, c-Jun, JunB and JunD from Santa Cruz Biotechnology. 

### 4.7. *In Vivo* Therapeutic Effect of Hippuristanol

Five-week-old female C.B-17/Icr-SCID mice, obtained from Kyudo, Co. (Tosu, Japan), were maintained in containment level 2 cabinets and provided with autoclaved food and water *ad libitum*. They were inoculated intraperitoneally with 5 × 10^6^ BCBL-1 cells and then randomly placed into two cohorts of five mice each that received vehicle or hippuristanol. Treatment was initiated on the day after cell injection. Hippuristanol was dissolved in 5.2% polyethylene glycol 400 (Wako Pure Chemical Industries) and 5.2% Tween 80 (Becton Dickinson, Franklin Lakes, NJ, USA) at a concentration of 0.25 mg/mL, and hippuristanol was administered at 7.5 mg/kg body weight every day for 35 days. Tumor burden was evaluated by measuring body weight. All mice were sacrificed on day 35, and then the liver, spleen and kidneys were dissected out and their weight was measured. Organ infiltration by BCBL-1 cells was evaluated by H&E staining. Treatment efficacy was determined by measuring the serum levels of human sCD30 by ELISA (BioVendor Inc., Brno, Czech Republic). This experiment was performed according to the guidelines for Animal Experimentation of the University of the Ryukyus and approved by the Animal Care and Use Committee of the same University. 

### 4.8. Statistical Analysis

All values are expressed as mean ± SD. Differences between groups and between treatments were tested for statistical significance by the Mann-Whitney *U*-test and Student’s *t*-test as appropriate. A confidence level of *P* < 0.05 was chosen as indication of statistical difference.

## 5. Conclusions

The present results indicated that hippuristanol is a potentially promising natural product for the treatment of PEL, warranting further exploration. Hippuristanol reduced cell viability through the induction of G_1_ cell cycle arrest and apoptosis, and these effects were mediated, at least in part, by inactivation of AP-1, STAT3 and Akt pathways.
